# High-Accuracy and High-Resolution Calorimetry Revealing New Correlations of Phase Change Enthalpy, Entropy, and Number of Carbon Atoms *n* in *n*-Alkanes

**DOI:** 10.3390/molecules30061300

**Published:** 2025-03-13

**Authors:** Harald Mehling, Jan Thoen, Christ Glorieux, Mary Anne White

**Affiliations:** 1 Consultant (R&D), Weingartenstr. 37, 97072 Würzburg, Germany; 2 Laboratory for Soft Matter and Biophysics, Department of Physics and Astronomy, KU Leuven, Celestijnenlaan 200D Box 2416, 3001 Leuven, Belgium; 3 Department of Chemistry, Clean Technologies Research Institute, Dalhousie University, 6274 Coburg Road, P.O. Box 15000, Halifax, NS B3H 4J3, Canada; mary.anne.white@dal.ca

**Keywords:** enthalpy, temperature, entropy, phase change, melting, crystal, phase change material, alkanes

## Abstract

Correlations between material properties are useful in engineering, and in addition, the underlying common mechanisms allow for a better understanding of the origins of the properties. Properties related to phase changes are an example, being important, e.g., in thermodynamic applications. For *n*-alkanes used, e.g., as phase change materials for thermal energy storage, linear correlations between enthalpy changes and entropy changes in phase transitions, as well as the number of carbon atoms *n*, have been observed and described by many researchers. Different correlations for odd and even *n* were found, though still with significant outliers. In this work, data from high-accuracy and high-resolution calorimetry were used for the analysis of enthalpy and entropy changes for alkanes with *n* = 14 to 30. The analysis shows more, and different, correlations than described in the previously published literature. Specifically, the ‘outliers’ have a physical and chemical origin, rooted in the phase transitions present in the specific *n*-alkanes, not just in whether *n* is odd or even. These detailed findings promise a better understanding of the thermodynamics of phase transitions.

## 1. Introduction

Changes in phase can be due to a change in physical composition (e.g., crystal or amorphous structure), chemical composition (e.g., molecular structure), or both. Thus, changes in phase are accompanied by changes in material properties, most significantly between solid and liquid and liquid and gas phases, and to a lesser degree between different solid phases. Phase transitions have an associated uptake or release of heat. Thus, phase changes are a topic of general interest in physics and chemistry and important in many technical areas, e.g., engineering of heat engines, or thermal energy storage (TES), often also called heat or cold storage depending on the application. For TES, the phase change most commonly used is between solid and liquid phases, but the transition between different solid phases is also possible. These phase changes typically occur in a narrow temperature range. If the phase change occurs at constant temperature, then the heat involved in the phase change is called “latent heat” (hidden heat), and the material is called a “latent heat storage material”, often even if the temperature is not constant. Generally, materials that store a significant amount of heat this way are called phase change materials (PCMs). Nowadays, PCMs are used in many commercial applications (Mehling et al. [1]), and the fields of application are growing. A well-known class of PCMs is that of the linear, saturated alkanes, also called *n*-alkanes. They are also employed in many other areas, and thus their properties are of general interest.

Knowledge of property values is crucial in engineering to select materials for designing an application. Commonly, values are tabulated, but if property value correlations exist, then these correlations allow for a more concise description, simply by a mathematical formula. The basis for the existence of correlations is an underlying common mechanism. Therefore, in addition to allowing for a simpler description by a mathematical formula, such correlations can allow for a deeper understanding of the origin of properties, including the values of the properties. Correlations, and the reasons for them, are thus highly interesting not only in engineering but also in physics and chemistry.

For PCMs, the most relevant properties are the temperature at which the phase change occurs and the enthalpy change during the phase change. Their values are commonly tabulated, but empirical correlations have also been derived, within a material class, and sometimes even across material classes.

Changes in enthalpy go along with changes in entropy. For a material undergoing phase change (a system), in thermal equilibrium at temperature *T* and pressure *p*, the Gibbs energy is at a minimum, such that Δ*G* = 0. Δ*G* is related to enthalpy *H* and entropy *S* by

(1) ΔG=ΔH−Δ(T·S).

Hence, and because the phases are in equilibrium at the same temperature *T*, it follows that

(2) ΔH=T·ΔS.

For molar values, the corresponding equation is modified by subscript m,

(3) $$\Delta H_{m}=T\cdot \Delta S_{m}.$$

In different phases of a substance, the relative position and motion of its particles, being atoms or molecules, are different. For molecules, the position and motion of the atoms they are made of are also included. A change from one phase to another causes related changes in entropy, internal energy, and enthalpy. The latter changes are closely connected with the (intra- and inter-) molecular interactions and thus can give insight into those interactions. Thus, within material classes, property correlations usually exist. Specifically, correlations should exist between the enthalpy change ∆*H*_m_ and the entropy change ∆*S*_m_, and analysis of their correlations promises a better understanding of material behavior. For the elements, a detailed analysis of correlations between molar enthalpy change and molar entropy change (calculated using phase change enthalpy and temperature employing Equation (3)) was carried out for the solid–liquid phase change by Mehling [2]. For elements, information is available on the atomic and molecular structure in the solid and liquid phase. A plot of molar enthalpy change versus molar entropy change shows that elements with similar structures and phase changes form groups. For atomic elements such as metals, noble gases, and C, Si, and Ge, where only interparticle bonds change, different enthalpy–entropy correlations were observed for specific bond types. For elements, analysis of such correlations has resulted in a better understanding of what happens upon melting. In follow-up publications, a large number of chemical compounds were analyzed [3], and the same was repeated for the liquid–gas phase change [4]. Due to the large number of materials investigated therein, information on the atomic and molecular structure in the different phases was rare. Thus, only general correlations were investigated, and no correlation to material structure was explored in detail.

**Figure 1:**
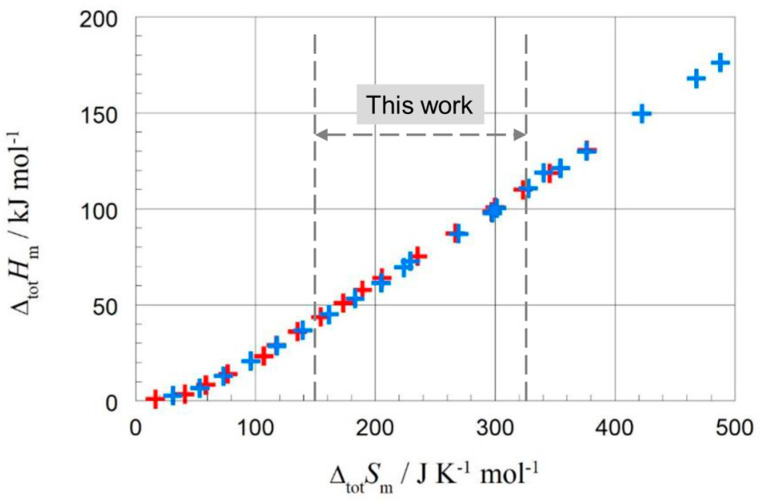
Molar total changes in enthalpy versus changes in entropy for alkanes; blue for even *n*, red for odd *n* (Mehling and White [7]). Span of present work is indicated.

**Figure 2:**
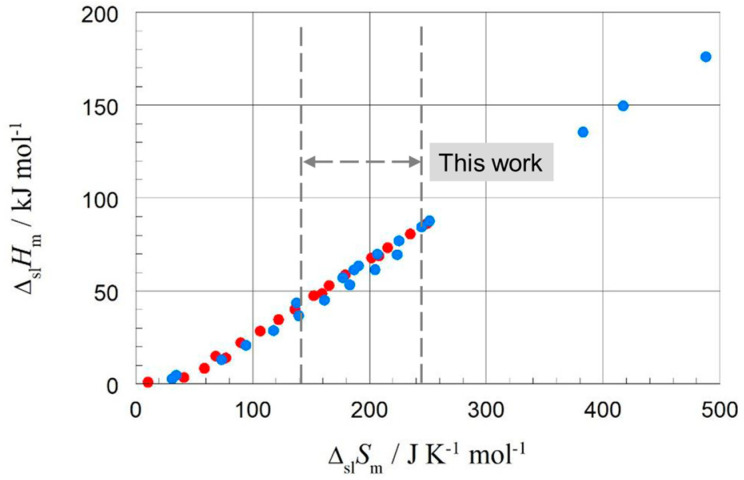
Molar solid–liquid changes in enthalpy versus changes in entropy for alkanes; blue for even *n*, red for odd *n* (Supplementary Materials in Mehling and White [7]). Span of present work is indicated.

Surveys on property–property and property–structure correlations of specific material classes have been performed by many researchers. For example, Dall’Acqua et al. [5] studied alkanes and alkane-α,ω-diamines, plotting melting temperature versus number of carbon atoms, *n*, molar melting enthalpy versus *n*, and molar melting entropy versus *n*. They found linear correlations between molar melting enthalpy and entropy for odd and even *n* separately. Their alkane study included 8 ≤ *n* ≤ 20. Badea et al. [6] studied odd–even effects in the melting properties of alkane-α,ω-diamides in similar plots, in comparison with alkane-α,ω-dioic acids and linear alkanes. For the alkanes, this was for 2 ≤ *n* ≤ 14. Mehling and White [7] studied alkanes, alcohols, and fatty acids, looking at data for the solid–liquid phase change, and solid–solid if applicable, as well as their total values (meaning the sum of the phase change enthalpy of solid–solid and solid–liquid phase changes and the same for entropy). The data used were compiled from three different literature sources. Correlations between enthalpy change and entropy change were investigated, as well as enthalpy change (and entropy change) and number of carbon atoms *n.* In many cases, linear correlations were observed, except for small *n*, with odd and even *n* often separate. For the linear alkanes C*_n_*H_2*n*+2_, the focus of the present work, the data available in the analysis in [7] were odd *n* = 1 to 35 and even *n* = 2 to 36, 40, 44, and 46. Figure 1 shows the enthalpy versus entropy for total values, and Figure 2 shows the same for solid–liquid values.

While the data showed linear correlations, they are not perfect, and for the solid–liquid phase change, some (but not all) data points for even *n* are nearly coincident with those for odd *n*, mainly in the range ∆_sl_*S*_m_ = 140 to 260 JK^−1^mol^−1^. This raises questions concerning separate linear correlations of even and odd *n*, as identified earlier. Specifically, are outliers due to real physical or chemical origin, or due to errors in the data?

In [7], as well as in investigations by other researchers, the data for a larger range of values of *n* come from literature data of phase change enthalpy and temperature. These data sets involve different sources for experimental values, and for each source, there are three issues:

1. The uncertainty of the individual measurement with respect to enthalpy change and temperature values. Often no uncertainty is presented, and even if it is present, it is often estimated too optimistically.
2. The way enthalpy and temperature values were determined from measurement data.
  For example, if DSC was used, a value for the phase change enthalpy can refer to the whole enthalpy change or only an integral above the peak baseline, and it can depend on the temperature range used for integration; similarly, the phase change temperature could be read from the peak onset or other points. A more detailed discussion can be found in [8,9]. The methods of determination are often not described in the literature, and different sources might also have used different methods.
3. The correct identification of the phase transition that the enthalpy change and temperature values refer to. For example, if DSC was used, high heating rates can cause a solid–solid and a solid–liquid phase change to overlap and look like a single phase change.

In the previous work by Mehling and White [7], based on other studies of phase change materials, it was assumed that uncertainties in phase transition temperatures are typical for DSC, namely less than 1.5 K, and uncertainties in transition enthalpy changes are less than 10%. These uncertainties are too large to resolve the above-mentioned questions. It would be preferable to have a single source of data for enthalpy and temperature values for a wide range of *n*, with known and small uncertainty, with the values determined in a consistent way from the measured data, and with measurements, etc., performed in such a way that the type of phase transition is correctly identified. For the *n*-alkane series of interest, we can make use of data that were all obtained with an adiabatic scanning calorimeter (ASC), which has been shown to provide high-accuracy and high-resolution measurements (Thoen et al. [10,11]).

The goal of this paper is to use the available data for *n*-alkanes, from high-accuracy and high-resolution measurements on very pure samples, and then re-evaluate enthalpy–entropy relationships and clarify the open questions with regard to correlations.

## 2. Materials and Methods

### 2.1. Materials

The data for the different transition temperatures and different heats of transitions for 17 *n*-alkanes (tetradecane [*n* = 14] to triacontane [*n* = 30]) have been obtained from extended high-resolution ASC measurements over the last 10 to 15 years. Some were published years ago or recently, while some have not been published previously. With regard to the specifics of materials, in all instances, products with the highest purity available were obtained from commercial suppliers. For all materials, with the exception of tetradecane, where the quoted purity was 99+%, the indicated purity (from gas chromatography) was higher than 99.5%. Further details can be found in the Supplementary Information (SI).

### 2.2. Methods

#### 2.2.1. Calorimetric Method to Determine *H*(*T*)

The calorimetric method used for the measurement of enthalpy as a function of temperature *H*(*T*) is a self-developed and -built adiabatic scanning calorimetry (ASC). Details of the calorimeter principle, setup, and operation can be found in the SI. Especially crucial with regard to the analysis of phase transitions are the accuracy of *H* and *T* and also the resolution in *T*. For the temperatures, an accuracy of ±0.2 K and a resolution of more than two orders of magnitude better are needed. For the specific enthalpy *H*(*T*), a standard uncertainty of 2% can be assigned, provided that the uncertainty on the sample mass is below this value, which is the case here; the resolution is one to two orders of magnitude better. This is much better than what is usually achieved with DSC, the method commonly used in R&D, for which uncertainties in phase transition temperatures are less than 1.5 K, and uncertainties in transition enthalpy changes are less than 10% [9].

#### 2.2.2. Determination of Transition Temperatures and Transition Heats

The method to determine the transition temperatures and transition heats from the measured *H*(*T*) curves is described in the corresponding section in the SI.

Altogether, the critical issues mentioned above with regard to the data basis are avoided here. The uncertainty of 2% for the individual measurement with respect to enthalpy change and temperature values (±0.2 K) is very small, as confirmed by testing. The method of the determination of enthalpy and temperature values for the transitions from measurement data is consistent throughout the whole data set. The high resolution in temperature allows for a clear separation of the different transitions and their individual identification.

#### 2.2.3. Calculation of the Data Basis

Using the enthalpy–temperature curve *H*(*T*) by ASC, the following steps were performed:

- The phase transitions (solid–solid and solid–liquid) were identified, and the corresponding enthalpy change (in J/g) and temperature (in K) for each transition were determined.
- The enthalpy change values were then converted from J/g to J/mol, using molar mass values.
- The entropy change was then calculated using Equation (3).
- For total values, enthalpy changes in solid–solid and solid–liquid transitions were added; the same was applied for entropy changes.

A note concerning notation: As per IUPAC recommendations [12], we express molar enthalpy changes as Δ*H*_m_ and molar entropy changes as Δ*S*_m_, and we use subscripts on Δ to indicate the type of change, i.e., Δ_ss_ for solid–solid transition, Δ_sl_ for solid–liquid transition (i.e., fusion or melting), and Δ_tot_ for total transition (ss + sl), where Δ_tot_*H*_m_ = Δ_sl_*H*_m_ + Δ_ss_*H*_m_ and Δ_tot_*S*_m_ = Δ_sl_*S*_m_ + Δ_ss_*S*_m_.

## 3. Results

### 3.1. Data Basis

Tables S1 and S2 of the Supplementary Information (SI) provide an overview of the data basis, comprising the number of carbon atoms, *n*, the chemical name, the molar mass, the measured transition temperature and enthalpy change in J/g, and the calculated values for the molar enthalpy change in J/mol and calculated molar entropy changes in JK^−1^mol^−1^. The values cover all *n*-alkanes from *n* = 14 to *n* = 30. Values from calorimetric measurements are partly from earlier measurements, published (Mehling et al. [8], Thoen et al. [13], Leys et al. [14]) as well as not yet published, and also new ones. The data relevant to the discussion are shown in Table 1. According to the measurement uncertainties, temperatures are given to 0.1 °C, and enthalpies and entropies are given as three digits. The full details are given in the SI.

### 3.2. Graphical Evaluation of Enthalpy Change vs. Entropy Change

This section presents the results for the graphical evaluation, meaning enthalpy change versus entropy change plots, for total values, solid–liquid transitions (i.e., fusion or melting), and solid–solid crystal–rotator transitions (order–disorder). A separate evaluation for solid–solid crystal–crystal transitions was not performed, as there are only two instances (*n* = 27 and 29).

#### 3.2.1. Total Values

**Figure 3:**
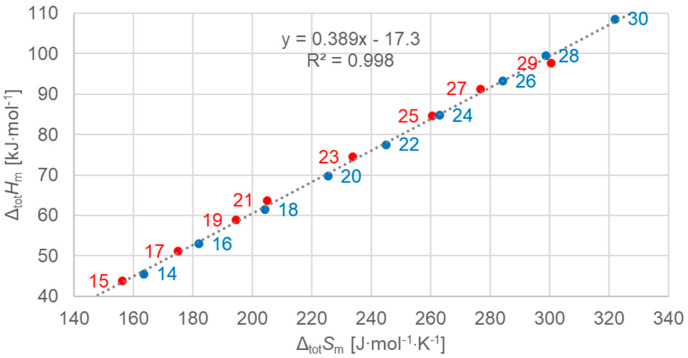
Molar total changes in enthalpy versus changes in entropy for *n*-alkanes investigated, all *n* (blue for even *n*, red for odd *n*).

Figure 3 shows the data points for the total values of all *n*, odd and even, and a linear fit to all the data points. The same color code is used: blue for even *n*, red for odd *n*. The colors apply to data and linear fits. And lines are dark grey if odd and even are observed together, e.g., for the linear fit to all data in Figure 3. Figure 3 shows the molar total changes in enthalpy versus changes in entropy. The linear fit to all *n* describes all data points well. The parameters of fit are given here, and later, with no more than three significant digits, in line with the uncertainties of enthalpy changes and entropy changes. Figure 3 confirms earlier observations, e.g., those from Figure 1 of [7]. Strikingly, the arrangement of the data points on the fitted line in Figure 3 shows that the data points of odd and even *n* are not located according to increasing *n*. Instead, even *n* points are shifted by one spot.

**Figure 4:**
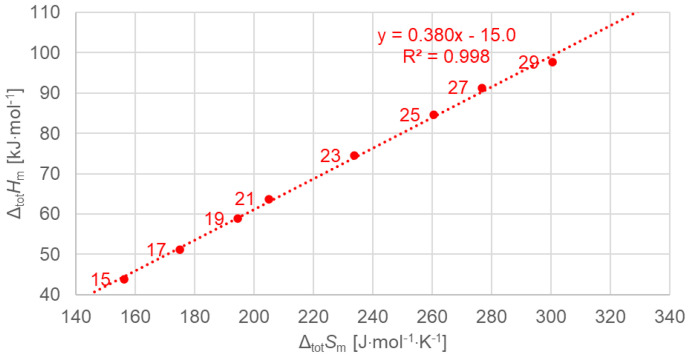
Molar total changes in enthalpy versus changes in entropy for *n*-alkanes investigated, odd *n* only.

Figure 4 shows the data points for the total values of odd *n*, now separately, and a linear fit to the data points. The linear fit describes all data points very well. This is not surprising, as the same was already observed for all *n*, even *n* and odd *n*, as shown in Figure 3.

**Figure 5:**
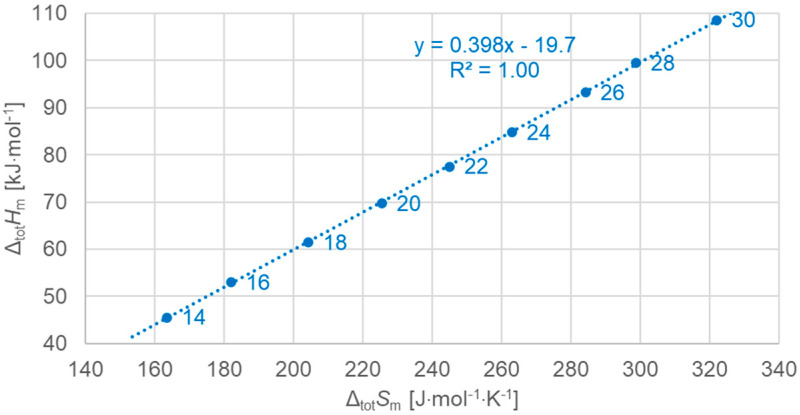
Molar total changes in enthalpy versus changes in entropy for *n*-alkanes, even *n* only.

Figure 5 shows the data points for the total values of even *n*, now separately, and a linear fit to the data points. The linear fit describes all data points perfectly. This is again not completely surprising looking at all *n*, even *n* and odd *n*, in Figure 3. However, while for odd *n* in Figure 4 there are small deviations from the linear fit, for even *n* in Figure 5, the linear correlation is perfect, as indicated by R^2^ = 1.00 (rounded to three digits).

#### 3.2.2. Solid–Liquid Values

**Figure 6:**
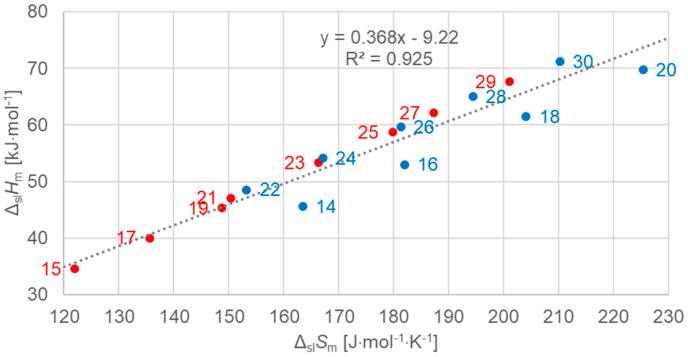
Molar solid–liquid changes in enthalpy versus changes in entropy for *n*-alkanes, all *n* (blue for even *n*, red for odd *n*).

Figure 6 shows the data points for the solid–liquid phase change for *n*-alkanes, odd and even *n*. A linear fit to all data points shows a correlation far from perfect. This is in accordance with earlier observations, e.g., in Figure 2, which was a reason for this more detailed analysis.

**Figure 7:**
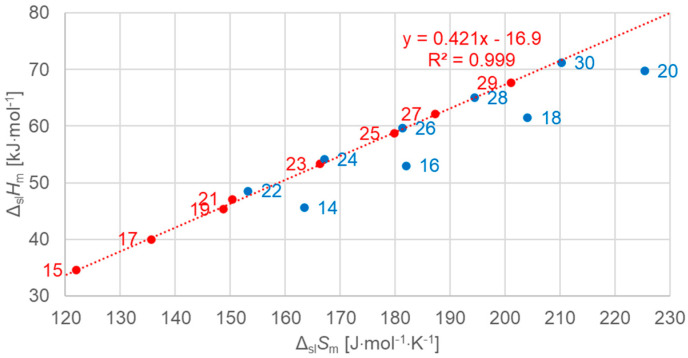
Molar solid–liquid changes in enthalpy versus changes in entropy for *n*-alkanes, red for odd *n* only (blue for even *n* for comparison).

Figure 7 shows the data points for the solid–liquid phase change, for odd *n* including a linear fit to the data points, and data points for even *n* separately for comparison. The data points for odd *n* are well described by the linear fit. This also holds for the data points for even *n* from *n* = 22 to 30, but not for *n* = 14, 16, 18, 20. As Table 1 shows, all data points that are described well by the linear fit have a solid–solid transition, while those that are not (*n* = 14, 16, 18, 20) have no solid–solid transition. This finding is in contrast to previous investigations, where linear correlations were stated for odd *n* as well as even *n*.

**Figure 8:**
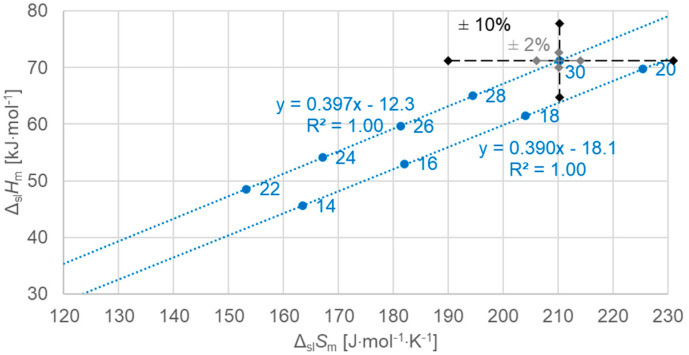
Molar solid–liquid changes in enthalpy versus changes in entropy for *n*-alkanes, even *n* only, where *n* is indicated. The alkanes with solid–solid transitions (upper line) were fitted separately from those with no solid–solid transitions (lower line).

Figure 8 shows the data points for the solid–liquid phase change for even *n*, now separate, and a linear fit to the data points for *n* = 14, 16, 18, 20, which have no solid–solid transition, and a separate fit for *n* = 22, 24, 26, 28, 30, which have a solid–solid transition. Again, the linear fits describe the data points perfectly, with an R^2^ = 1.00 (rounded to three digits).

The importance of using high-resolution and high-accuracy data here becomes clear by the marks on the *n* = 30 data point in Figure 8, which indicate two ranges of uncertainty, ±10% and ±2%. Only by the smaller uncertainty here, compared to literature data, is it possible to resolve the subtle details that are observed here. It is important to note that, as Δ*S* is derived from *T* and Δ*H* by Equation (3), and because the uncertainty in *T* is negligible, the uncertainties in Δ*S* and Δ*H* are practically the same. Moreover, their deviations go in the same direction. As a consequence, a deviation in the measured Δ*H* leads to a roughly diagonal shift in Figure 8. This is crucial when discussing the order of *n* along the linear fit lines.

#### 3.2.3. Solid–Solid Values (Plots Only for Order–Disorder Transitions [od], Meaning Ordered Crystal to Rotator Phase)

**Figure 9:**
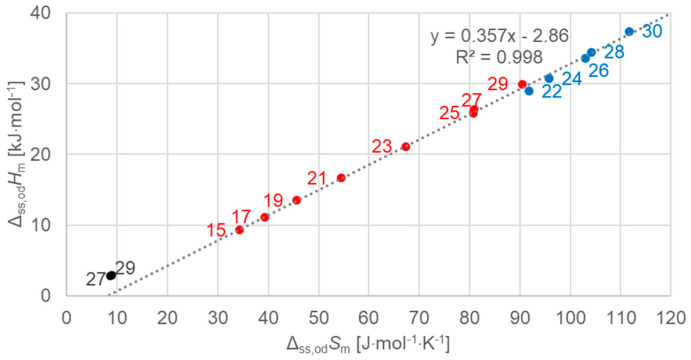
Molar solid–solid (crystal–rotator) changes in enthalpy versus changes in entropy for *n*-alkanes, all *n* (blue for even *n*, red for odd *n*). Also included, in black, are solid–solid crystal–crystal transitions for *n* = 27 and 29.

Figure 9 shows the data points for all *n*-alkanes investigated that undergo solid–solid crystal–rotator phase changes, for all *n*, odd and even, and a linear fit to all the data points. Again, the linear fit describes these data points quite well. It is striking that the data points are not located in a continuous series with changing *n*. While for the total values in Figure 3 an offset by one in terms of *n* is observed, here, for solid–solid, the shift is even more pronounced.

In addition, also shown are two data points for solid–solid crystal–crystal transitions of *n* = 27 and 29 (see Table 1). A separate evaluation of solid–solid crystal–crystal transitions was not performed, because there are only two instances. Figure 9, however, already shows that these transitions have far lower enthalpy and entropy changes than those of solid–solid crystal–rotator phase changes. The data for *n* = 27 and 29 are closer to those of lower *n* than expected, and thus the observed shift in odd *n* of solid–solid (crystal–rotator) changes in Figure 9 might be correlated with the existence of solid–solid crystal–crystal transitions for *n* = 27 and 29.

**Figure 10:**
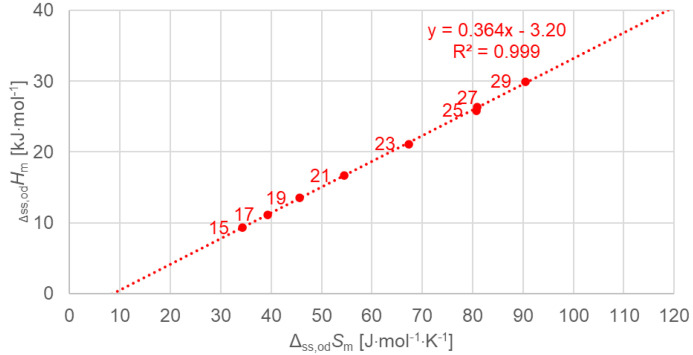
Molar solid–solid (crystal–rotator) changes in enthalpy versus changes in entropy for *n*-alkanes, odd *n* only.

Figure 10 shows the data points for the solid–solid crystal–rotator phase change, for odd *n* separately, including a linear fit to the data points. Again, the linear fit describes the data points very well.

**Figure 11:**
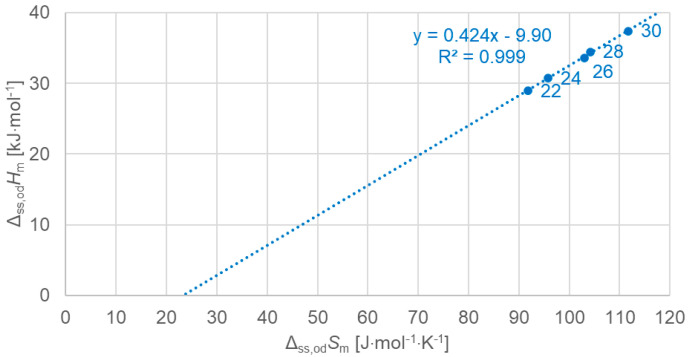
Molar solid–solid (crystal–rotator) changes in enthalpy versus changes in entropy for *n*-alkanes, even *n* only.

Figure 11 shows the data points for the solid–solid crystal–rotator phase change, for even *n* separately, including a linear fit to the data points. Again, the linear fit describes the data points very well.

### 3.3. Graphical Evaluation of Enthalpy vs. n and Entropy vs. n

This section presents the results of the graphical evaluation of enthalpy change versus *n* and entropy change versus *n* for total values, solid–liquid transitions (i.e., fusion or melting), and solid–solid crystal–rotator transitions (order–disorder). A separate evaluation for solid–solid crystal–crystal transitions was not performed, as there are only two instances.

#### 3.3.1. Total Values

**Figure 12:**
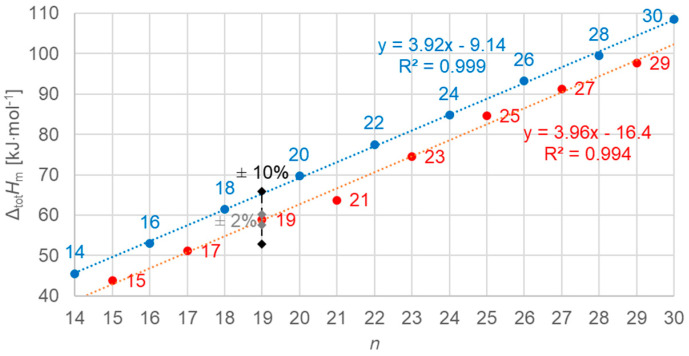
Molar total changes in enthalpy versus *n* for *n*-alkanes, all *n* (blue for even *n*, red for odd *n*). Again, high-resolution data (±2%) are required to discern trends not seen in less accurate data (e.g., ±10%, as for DSC).

Figure 12 shows the data points for the total values of molar enthalpy change versus *n*, all *n*, odd and even. Obviously, there is no linear correlation for all data; however, there is an excellent correlation for odd *n* and even *n* separately, as the line fits show. The importance of using high-resolution and high-accuracy data here becomes clear again by the marks on the *n* = 19 data point, which indicate the range of ±10% and ±2% uncertainty. Only by the smaller uncertainty here, compared to common literature data, is it possible to resolve the details that are observed here.

**Figure 13:**
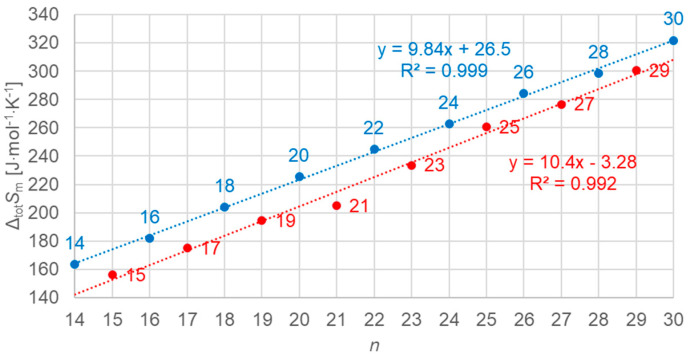
Molar total changes in entropy versus *n* for *n*-alkanes, all *n* (blue for even *n*, red for odd *n*).

Figure 13 shows the data points for the total values of molar entropy change versus *n*, all *n*, odd and even. As for enthalpy change versus *n*, there is a separate linear correlation for odd *n* and even *n*.

#### 3.3.2. Solid–Liquid Values

**Figure 14:**
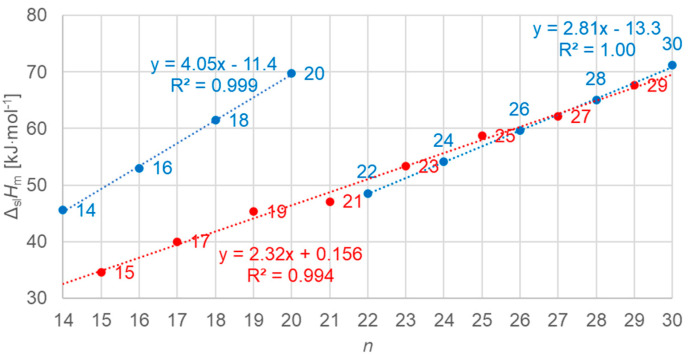
Molar solid–liquid changes in enthalpy versus *n* for *n*-alkanes, all *n* (blue for even *n*, red for odd *n*).

Figure 14 shows the data points for the solid–liquid values of molar enthalpy change in alkanes versus *n*, all *n*, odd and even. Obviously, there is no linear correlation for all the data; however, there is a good one for odd *n* alone, and very good ones separately for even *n* with *n* = 14 to 20 (no solid–solid transition) and for *n* = 22 to 30 (also have a solid–solid transition). This again shows that *n* being odd or even is not the main determinant of the behavior, but instead the types of transitions present altogether in an *n*-alkane.

**Figure 15:**
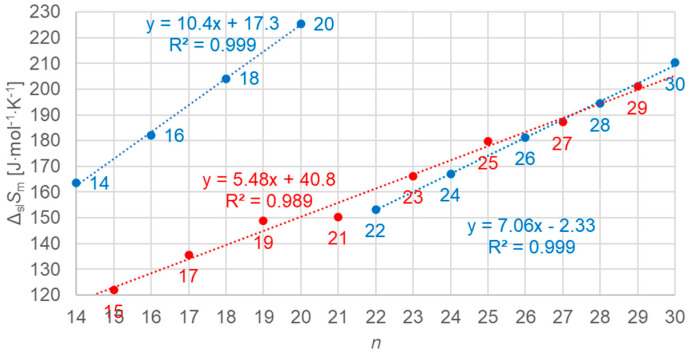
Molar solid–liquid changes in entropy versus *n* for *n*-alkanes, all *n* (blue for even *n*, red for odd *n*).

Figure 15 shows the data points for the solid–liquid values of molar entropy changes for *n*-alkanes versus *n*, all *n*, odd and even. As before for enthalpy change versus *n*, for entropy change versus *n*, a similar behavior is observed.

#### 3.3.3. Solid–Solid Values

**Figure 16:**
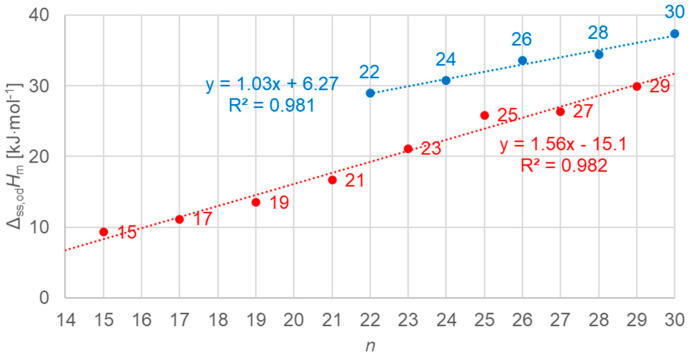
Molar solid–solid (crystal–rotator) changes in enthalpy versus *n* for *n*-alkanes, all *n* (blue for even *n*, red for odd *n*).

Figure 16 shows the data points for the solid–solid (crystal–rotator) values of molar enthalpy change versus *n*, all *n*, odd and even. There is a linear correlation for all odd *n* data, and another for even *n* with *n* = 22 to 30; *n* = 14 to 20 have no solid–solid transition.

**Figure 17:**
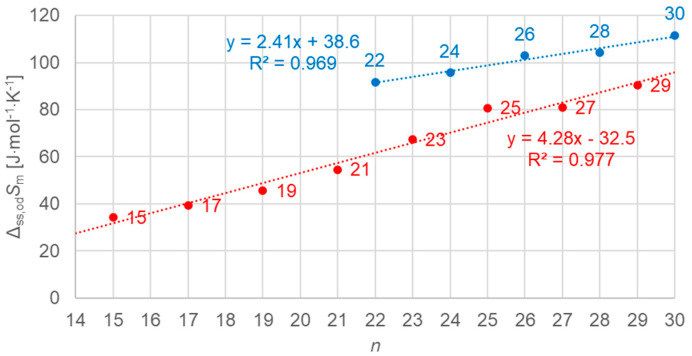
Molar solid–solid (crystal–rotator) changes in entropy versus *n* for *n*-alkanes, all *n* (blue for even *n*, red for odd *n*).

Figure 17 shows the data points for the solid–solid (crystal–rotator) values of molar entropy change versus *n*, all *n*, odd and even. As for enthalpy versus *n*, a similar behavior is observed.

## 4. Discussion

As in previous investigations by many authors, here too were linear correlations observed for enthalpy change versus entropy change, enthalpy change versus *n*, and entropy change versus *n*. However, the new observations made here add details and also reveal significant deviations. While Mehling and White [7] observed deviations from linear correlations for small values of *n* (Figure 1 and Figure 2), here we highlight details concerning higher values of *n* (*n* = 14 to 30).

Previous investigations stated linear correlations for all *n*, sometimes for odd *n* and for even *n* separately. However, here it was found that the odd or even character of *n* is not the main influence on the correlations. Instead, here, all involved types of transitions turn out to be of importance. In the group of *n*-alkanes investigated here, from *n* = 14 to 30, all have a solid–liquid transition, and all except *n* = 14, 16, 18, and 20 also have a solid–solid crystal–rotator transition, and *n* = 27 and 29 additionally have a solid–solid crystal–crystal transition. In the enthalpy change versus entropy change plots, the influence of other transitions is obvious, looking at the data of solid–liquid transitions (Figure 6, Figure 7 and Figure 8); for solid–solid transitions, it is also seen to a small degree (Figure 9, Figure 10 and Figure 11). However, looking at the total values (Figure 3, Figure 4 and Figure 5), it is not seen at all. Deviating even further from the common statement that correlations depend on odd or even *n* are observations of enthalpy change versus *n* and entropy change versus *n*. The high-accuracy and high-resolution data used here reveal more deviations. For enthalpy change versus *n*, solid–liquid transitions with odd *n* and even *n* even show different linear correlations for those *n*-alkanes that also exhibit solid–solid transitions (Figure 14 and Figure 15), and for entropy change versus *n*, this is also seen for solid–solid transitions (Figure 16 and Figure 17). On the contrary, for the total values of odd *n* or even *n*, the value of *n* is the main factor (Figure 12 and Figure 13).

Another type of deviation refers to the order of *n* along linear correlations. In this work, *n* values are included in all graphs. For enthalpy versus entropy correlations, Figure 3, showing the total values of all *n*, already shows an offset of *n* between odd and even by 1 (the order along the linear fit is *n* = 15, 14, 17, 16, …). Offsets are also present in the solid–liquid values (Figure 6), and strongest in the solid–solid values (Figure 9). This presentation points to the solid–solid transition enthalpy and entropy being highly dependent on whether *n* is odd or even. Additionally, while these solid–solid transitions are crystal–rotator transitions, the crystal–crystal transitions of *n* = 27 and 29 show again a different behavior (Figure 9).

Altogether, using data from high-accuracy and high-resolution measurements here, with an uncertainty far lower than common in the literature, results in two achievements. First, regarding the initial goal of this research, it is shown that outliers from linear correlations, observed in previous investigations, are not merely associated with uncertainties or errors in the data. It is now clear that there is a real physical or chemical origin. Second, the origin is identified as far as it is clearly related to the types of transitions present.

That these achievements are not possible with common literature data is briefly shown in the SI (Figures S3–S6). These figures show exemplary graphs of enthalpy versus n and entropy versus n using the common literature data from [7], originally from [15,16,17], instead of using the present high-accuracy and high-resolution data as shown above. In the SI figures it is not possible to see the newly discovered correlations. In common literature data the typical scattering in data of about 10% in enthalpy (for octadecane analyzed in detail by [18]) does not show sufficient details. With high-accuracy and high-resolution data, as used here, more detailed correlations can be revealed, and more research can be done in future.

Future research, as a consequence, should identify in more detail the physical and chemical origins. As the data basis available up to now is *n* = 14 to 30, additional measurements should be made to extend the existing data basis to both smaller and larger *n*. Last but not least, it must be expected that similar observations can be made by looking at other material classes, such as the alkanols or fatty acids. Looking at more material classes promises a much deeper understanding of the thermodynamics of phase changes.

## Figures and Tables

**Table 1:** Overview of all data relevant to the discussion, sorted by *n* odd and *n* even.

				Solid–Solid cc (Crystal–Crystal)			Solid–Solid od (Crystal–Rotator)			Solid–Liquid (Fusion)			Total (Sum of All)	
Odd/Even	n (C…)	Name	Molar Mass [g∙mol^−1^]	*T*_ss_ [K]	Δ_ss_*H*_m_ [kJ∙mol^−1^]	Δ_ss_*S*_m_ [J∙mol^−1^∙K^−1^]	*T*_ss_ [K]	Δ_ss_*H*_m_ [kJ∙mol^−1^]	Δ_ss_*S*_m_ [J∙mol^−1^∙K^−1^]	*T*_sl_ [K]	Δ_sl_*H*_m_ [kJ∙mol^−1^]	Δ_sl_*S*_m_ [J∙mol^−1^∙K^−1^]	Δ_tot_*H*_m_ [kJ∙mol^−1^]	Δ_tot_*S*_m_ [J∙mol^−1^∙K^−1^]
even	14	n-tetradecane	198.39							278.8	45.6	163	45.6	163
even	16	n-hexadecane	226.44							291.1	53.0	182	53.0	182
even	18	n-octadecane	254.49							301.2	61.5	204	61.5	204
even	20	n-eicosane	282.55							309.5	69.7	225	69.7	225
even	22	n-docosane	310.60				315.9	29.0	91.7	316.8	48.5	153	77.5	245
even	24	n-tetracosane	338.65				321.2	30.7	95.7	323.7	54.1	167	84.8	263
even	26	n-hexacosane	366.71				326.2	33.6	103	329.1	59.7	181	93.3	284
even	28	n-octacosane	394.76				330.7	34.5	104	334.3	65.0	194	99.4	299
even	30	n-triacotane	422.82				335.2	37.4	112	338.5	71.2	210	109	322
odd	15	n-pentadecane	212.41				270.8	9.3	34.3	283.0	34.5	122	43.8	156
odd	17	n-heptadecane	240.47				284.1	11.2	39.3	294.9	40.0	136	51.1	175
odd	19	n-nonadecane	268.52				295.8	13.5	45.7	304.9	45.4	149	58.9	194
odd	21	n-heneicosane	296.57				305.2	16.6	54.5	313.1	47.1	150	63.7	205
odd	23	n-tricosane	324.63				313.7	21.1	67.4	320.6	53.3	166	74.4	234
odd	25	n-pentacosane	352.68				320.4	25.9	80.7	326.7	58.7	180	84.6	260
odd	27	n-heptacosane	380.73	323.1	2.78	8.60	326.3	26.4	80.9	331.9	62.1	187	91.3	277
odd	29	n-nonacosane	408.79	326.0	2.90	8.90	331.4	30.0	90.4	336.5	67.7	201	97.6	300

## Data Availability

The original contributions presented in this study are included in the article/Supplementary Materials. Further inquiries can be directed to the corresponding author.

## Supplementary Materials

The following supporting information can be downloaded at: https://www.mdpi.com/article/10.3390/molecules30061300/s1, Detailed information on the materials, calorimeter, determination of values as well as accuracy and resolution, and the complete data basis. References [7,8,11,13,14,15,16,17,18,19,20,21,22,23] are cited in the Supplementary file.
