# Supplementary material for: High-Accuracy and High-Resolution Calorimetry Revealing New Correlations of Phase Change Enthalpy, Entropy, and Number of Carbon Atoms n in n-Alkanes
Source: Molecules. 2025 Mar 13;30(6):1300. doi: 10.3390/molecules30061300 (PMC11945070; doi:10.3390/molecules30061300)
Supplement: Supplementary file 1 [file molecules-30-01300-s001.zip › molecules-3411906-supplementary.pdf]

High-Accuracy and High-Resolution Calorimetry Revealing New Correlations of Phase Change Enthalpy, Entropy, and Number of Carbon Atoms *n* in n-Alkanes

Supplementary Materials

Harald Mehling [1], Jan Thoen [2], Christ Glorieux [2] and Mary Anne White [3]

[1] Consultant (R&D), Weingartenstr. 37, 97072 Würzburg, Germany

[2] Laboratory for Soft Matter and Biophysics, Department of Physics and Astronomy, KU Leuven, Celestijnenlaan 200D Box 2416, 3001 Leuven, Belgium

[3] Department of Chemistry and Clean Technologies Research Institute , Dalhousie University, 6274 Co-burg Road, P.O. Box 15000, Halifax, NS B3H 4J3, Canada

Correspondence: harald.mehling@gmail.com

This supplementary information contains all details that are of interest, but not crucial for the main text. Specifically, this comprises details on the materials investigated, a description of the calorimeter used, incl. accuracy and resolution, and finally the data basis used in the evaluation collected in two tables.

1 Data basis

1.1 Materials

The data for the different transition temperatures and different heats of transitions for 17 *n*-alkanes (tetradecane n = 14 to triacontane n = 30) have been obtained from extended high-resolution p-ASC (see below) measurements over the last 10 to 15 years. At all instances, materials with the highest purity available have been obtained from commercial suppliers. For all, with the exception of tetradecane (from Aldrich) where the quoted purity was 99+ %, the indicated purity (from gas chromatography) was better than 99.5 %. For pentadecane to eicosane more details on the suppliers and purity can be found in [13]. Docosane and tetracosane with purities of 99.9 %, were purchased from Supelco. For heneicosane (from Fluka) and for triacosane the quoted purities were 99.7 % and 99.8 %, respectively. The products pentacosane to triacontane all came from Fluka with a quoted purity of >99.5 %.

1.2 Description of the calorimeter and accuracy and resolution tests

Calorimeter principle, setup, and operation

In an adiabatic calorimeter the sample is ideally in an adiabatic environment, and heated electrically via an electrical resistance heater. From the energy supplied electrically, which is equal to the heat supplied, and the change of the sample temperature, the temperature dependence of the enthalpy and of the heat capacity can be calculated. The adiabatic environment is realized by one or several temperature shields surrounding the sample in a vacuum chamber, of which the inner shield is kept at the same temperature of the sample and thereby assuring that there is no temperature difference with the sample, thus no heat exchange. Adiabatic calorimeters are known for high accuracy. Different modifications exist, to measure in steps or scanning a temperature range, as well as allowing for small heat exchange with the environment and thereby also measurements on cooling.

All enthalpy curves were obtained by a lab-built Peltier-element-based adiabatic scanning calorimeter (pASC) of which a schematic representation is given in Figure S1. In this approach, the crucible, which contains the sample material, is placed on a thin metal plate platform together with a resistor for supplying a constant heating power P and a calibrated resistance thermometer for measuring the temperature *T* of the sample by means of a Keithley 2010 multimeter with scanner card 2000. A source meter Keithley 2400 is not only supplying a programmed power to the heating resistor on the platform, but it is also measuring it precisely, allowing to know $P(t)$ at all times precisely in spite of a (small) change of the heating resistor with temperature. Both the temperature and the power are measured at intervals of about 5 seconds. Typical power values are 100 to 500 microwatt. For typical sample sizes (a few tens of milligrams) these power values result outside phase transition ranges in scanning rates of tens of millikelvin per minute. In phase transition ranges the rates substantially decrease and reduce to zero during first-order phase transitions, the temperature staying constant until the necessary heat is delivered to cross the transition. This allows measurements with very high resolution in temperature.

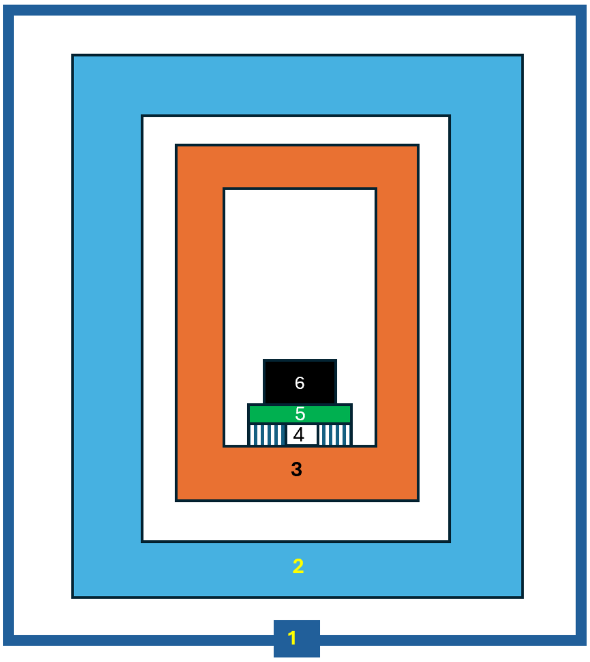

Figure S1. Schematic layout of pASC calorimeter.

From outside towards inside/down to up:

1. Temperature chamber, controlled to maintain a fixed temperature difference with Sh2
2. Shield 2, provided with R_{TSh2}, R_{PSh2}, controlled to maintain a fixed temperature difference with Sh1
3. Shield 1, provided with R_{TSh1}, R_{PSh1}, controlled to maintain $V_{Peltier}$=0
4. Peltier element, used to maintain $T_{platform}$-T_{Sh1}=0 via measurement of $V_{Peltier}$
5. Platform supporting the crucible, provided with $R_{Tsample}$ and $R_{Psample}$
6. Airtight crucible containing the sample

All empty space is kept vacuum.

The determination of the experimental (sample plus addenda) enthalpy curve E_{tot}(T) is straightforward: given the measured power $P(t)$ and platform temperature $T(t)$, its evolution is determined as $E_{tot}(t)=\int P(t)dt$. Combining with $T(t)$, $E_{tot}(T)$ is then known. It should be mentioned that:

(i) The obtained $E_{tot}(T)$ (unit joule) is the sum of the enthalpy of the sample and of the addenda: the crucible, the platform, the heating resistor and the thermometer. This implies that prior to determining

the enthalpy of the sample $E(T)$, a calibration needs to be performed of the enthalpy curve of the addenda without sample.

(ii) The construction and mode of operation of the calorimeter are such that no heat is flowing from the platform to its environment. This is ensured by keeping vacuum around the platform and by keeping the nearest environment of the platform, which we here refer to as shield 1, at the same temperature as the platform: zeroing the temperature difference $T_{platform}$-T_{Sh1} guarantees that the process is adiabatic and that all heat supplied via the heating resistor is staying in the platform sample combination. This zeroing is done by a PID loop that sends electric heating power to resistive heaters around shield 1 such that the voltage that is generated by a Peltier element, used as thermopile that is sandwiched between the platform and shield 1, is kept zero. Shield 1 is passively cooled by small thermal losses to shield 2, which surrounds shield 1. In practice the Peltier voltage can be kept below 1 microvolt, corresponding to less than 1 microwatt of power exchange between the sample and the environment via the Peltier element. Since shield 1 is made of massive copper and is completely surrounding the platform, its temperature is quite uniform, and thus near the platform and sample temperature across all its surfaces, ensuring minimum radiative heat loss and thus adiabatic conditions.

In the used calorimeter, cooling runs were performed by programming the PID control such that a constant Peltier voltage offset was kept at all times, with the temperature of shield 1 slightly colder than the platform. No current was sent through the heating resistor. Since the Peltier offset voltage is proportional to the cooling power that is flowing through the Peltier from the sample to the shield, with a quite temperature independent (calibrated) proportionality factor, this approach corresponds with a situation of a rather constant cooling power.

The slow scanning rates used and the very frequent (about every 5 seconds) temperature and power measurements, result in large data sets (several tens of thousands of t, T and P values), yielding almost continuous $E(T)$ (unit J) curves. This situation allows for accurate numerical differentiation of $E(T)$ resulting in heat capacity $C_p(T)$ = dE/dT (unit J/K) curves. Combinations with sample mass and molecular mass allow calculation of the specific or molar quantities of enthalpy and heat capacity.

Further details on the construction and operation of a Peltier-element-based adiabatic scanning calorimeter can be found in Thoen et al. [19], Leys et al. [20], Thoen et al. [11].

Determination of transition temperatures and transition heats

In case of a phase transition, the transition heat was determined by performing linear regression of $H(T)$ well below and well above the transition temperature range. The regression lines were then extrapolated, respectively upwards and downwards, till the phase transition temperature T_{pc}. The difference between the extrapolated lines evaluated at T=T_{pc} was used as the transition heat. See Figure S2.

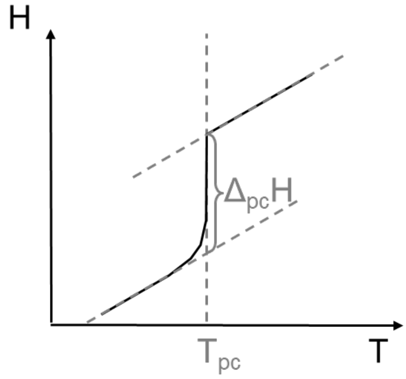

Figure S2. Schematic *H*-*T* graph, with regression lines for determination of T_{pc} and $\Delta_{pc}H$.

The value of T_{pc} itself was obtained by determining the intersection point of the regression line of $H(T)$ in the high-temperature phase with the fitting line of the linear part of $H(T)$ in the two-phase region.

Accuracy of the data

The uncertainty on the measured values of T(t), P(t), on the heat capacity of the empty calorimeter, on the heat capacity of a small amount of thermal paste (used for good thermal contact between the cell and platform), and on the sample mass, determine the accuracy of the final enthalpy and heat capacity results as well as on the heat of the transitions. These background heat capacities have been carefully measured in separate runs.

An important aspect (often overlooked) is the proper establishment of the temperature scale of the calorimeter. For the used pASC instrument, the thermistor thermometers (at the platform and at the different shields) were carefully calibrated in situ by means of a Tinsley Pt reference thermometer, previously calibrated within 0.003 K against the international temperature scale by the National Physical Laboratory (UK). For the in situ calibration a temperature controlled oven with a stability of 0.1 K was used. For the temperatures obtained in the calorimeter we arrived at an accuracy of ± 0.2 K. However, the resolution for the temperature is more than two orders of magnitude better.

On the basis of an extensive error analysis by Leys et al. [21], it could be concluded that a standard uncertainty of 2% can be assigned to the (specific) heat capacity and to the (specific) enthalpy, provided the uncertainty on the sample mass is below this value, which is the case here. It should be noted that the resolution (in particularly relevant for phase transitions) is one to two orders of magnitude better.

1.3 Data basis

Table S1 and Table S2 give an overview on the data basis, comprising the number of carbon atoms, *n*, the chemical name, the molar weight, the measured transition temperature and enthalpy change in J/g, and then calculated values for the molar enthalpy change in J/mol and calculated molar entropy changes. Table S1 gives the data sorted by *n*, Table S2 gives the data grouped by odd and by even as used in the discussion. As in the main text, and in [7], a consistent color code is used: blue for even *n*, red for odd *n*. Enthalpies in J/g, resulting directly from measurement but not needed later, are marked in light grey. As specified above, the uncertainties for the calorimetric measurements are ± 0.2 K in the temperature and 2% for (specific) heat capacity and (specific) enthalpy. Accordingly, the temperatures in Table S1 and Table S2 are given to 0.1°C, and the enthalpy and entropy with 3 digits.

Results are partly from earlier measurements, published as well as not yet published, and also new ones. Values for *n* = 14 were published previously in Mehling et al. [8]. Values for *n* = 15 to 20 were recently published in Thoen et al. [13]. Values for *n* = 21 and 22 are from new measurements for this publication. Values for *n* = 23 to 25 are from Leys et al. [14]. Values for *n* = 26 are again from new measurements. Values for *n* = 27 to 30 are from previous measurements, again, but have not been published until now. Values of molar mass are from the NIST data bank [22], except the one for *n* = 30 which was from VWR [23].

Table S1 Data sorted by *n*.

				solid-solid cc (crystal-crystal)				solid-solid od (crystal-rotator)				solid-liquid (fusion)				total (sum of all)			
odd / even	n (C...)	name	molar mass [g·mol^{-1}]	T_{ss} [K]	$\Delta_{ss}H$ [J·g^{-1}]	$\Delta_{ss}H_m$ [kJ·mol^{-1}]	$\Delta_{ss}S_m$ [J·mol^{-1}·K^{-1}]	T_{ss} [K]	$\Delta_{ss}H$ [J·g^{-1}]	$\Delta_{ss}H_m$ [kJ·mol^{-1}]	$\Delta_{ss}S_m$ [J·mol^{-1}·K^{-1}]	T_{sl} [K]	$\Delta_{sl}H$ [J·g^{-1}]	$\Delta_{sl}H_m$ [kJ·mol^{-1}]	$\Delta_{sl}S_m$ [J·mol^{-1}·K^{-1}]	T_{tot} [K]	$\Delta_{tot}H$ [J·g^{-1}]	$\Delta_{tot}H_m$ [kJ·mol^{-1}]	$\Delta_{tot}S_m$ [J·mol^{-1}·K^{-1}]
even	14	n-tetradecane	198.39									278.8	230	45.6	163	-	230	45.6	163
odd	15	n-pentadecane	212.41					270.8	43.7	9.28	34.3	283.0	163	34.5	122	-	206	43.8	156
even	16	n-hexadecane	226.44									291.1	234	53.0	182	-	234	53.0	182
odd	17	n-heptadecane	240.47					284.1	46.4	11.2	39.3	294.9	166	40.0	136	-	213	51.1	175
even	18	n-octadecane	254.49									301.2	242	61.5	204	-	242	61.5	204
odd	19	n-nonadecane	268.52					295.8	50.3	13.5	45.7	304.9	169	45.4	149	-	219	58.9	194
even	20	n-eicosane	282.55									309.5	247	69.7	225	-	247	69.7	225
odd	21	n-heneicosane	296.57					305.2	56.1	16.6	54.5	313.1	159	47.1	150	-	215	63.7	205
even	22	n-docosane	310.60					315.9	93.3	29.0	91.7	316.8	156	48.5	153	-	250	77.5	245
odd	23	n-tricosane	324.63					313.7	65.1	21.1	67.4	320.6	164	53.3	166	-	229	74.4	234
even	24	n-tetracosane	338.65					321.2	90.8	30.7	95.7	323.7	160	54.1	167	-	251	84.8	263
odd	25	n-pentacosane	352.68					320.4	73.3	25.9	80.7	326.7	167	58.7	180	-	240	84.6	260
even	26	n-hexacosane	366.71					326.2	91.6	33.6	103	329.1	163	59.7	181	-	254	93.3	284
odd	27	n-heptacosane	380.73	323.1	7.30	2.78	8.60	326.3	69.3	26.4	80.9	331.9	163	62.1	187	-	240	91.3	277
even	28	n-octacosane	394.76					330.7	87.3	34.5	104	334.3	165	65.0	194	-	252	99.4	299
odd	29	n-nonacosane	408.79	326.0	7.10	2.90	8.90	331.4	73.3	30.0	90.4	336.5	166	67.7	201	-	246	100	300
even	30	n-triacotane	422.82					335.2	88.5	37.4	112	338.5	168	71.2	210	-	257	109	322

Table S2 Data sorted by *n* odd and *n* even.

				solid-solid cc (crystal-crystal)				solid-solid od (crystal-rotator)				solid-liquid (fusion)				total (sum of all)			
odd / even	n (C...)	name	molar mass [g·mol^{-1}]	T_{ss} [K]	$\Delta_{ss}H$ [J·g^{-1}]	$\Delta_{ss}H_m$ [kJ·mol^{-1}]	$\Delta_{ss}S_m$ [J·mol^{-1}·K^{-1}]	T_{ss} [K]	$\Delta_{ss}H$ [J·g^{-1}]	$\Delta_{ss}H_m$ [kJ·mol^{-1}]	$\Delta_{ss}S_m$ [J·mol^{-1}·K^{-1}]	T_{sl} [K]	$\Delta_{sl}H$ [J·g^{-1}]	$\Delta_{sl}H_m$ [kJ·mol^{-1}]	$\Delta_{sl}S_m$ [J·mol^{-1}·K^{-1}]	T_{tot} [K]	$\Delta_{tot}H$ [J·g^{-1}]	$\Delta_{tot}H_m$ [kJ·mol^{-1}]	$\Delta_{tot}S_m$ [J·mol^{-1}·K^{-1}]
even	14	n-tetradecane	198.39									278.8	230	45.6	163	-	230	45.6	163
even	16	n-hexadecane	226.44									291.1	234	53.0	182	-	234	53.0	182
even	18	n-octadecane	254.49									301.2	242	61.5	204	-	242	61.5	204
even	20	n-eicosane	282.55									309.5	247	69.7	225	-	247	69.7	225
even	22	n-docosane	310.60					315.9	93.3	29.0	91.7	316.8	156	48.5	153	-	250	77.5	245
even	24	n-tetracosane	338.65					321.2	90.8	30.7	95.7	323.7	160	54.1	167	-	251	84.8	263
even	26	n-hexacosane	366.71					326.2	91.6	33.6	103	329.1	163	59.7	181	-	254	93.3	284
even	28	n-octacosane	394.76					330.7	87.3	34.5	104	334.3	165	65.0	194	-	252	99.4	299
even	30	n-triacotane	422.82					335.2	88.5	37.4	112	338.5	168	71.2	210	-	257	109	322
odd	15	n-pentadecane	212.41					270.8	43.7	9.3	34.3	283.0	163	34.5	122	-	206	43.8	156
odd	17	n-heptadecane	240.47					284.1	46.4	11.2	39.3	294.9	166	40.0	136	-	213	51.1	175
odd	19	n-nonadecane	268.52					295.8	50.3	13.5	45.7	304.9	169	45.4	149	-	219	58.9	194
odd	21	n-heneicosane	296.57					305.2	56.1	16.6	54.5	313.1	159	47.1	150	-	215	63.7	205
odd	23	n-tricosane	324.63					313.7	65.1	21.1	67.4	320.6	164	53.3	166	-	229	74.4	234
odd	25	n-pentacosane	352.68					320.4	73.3	25.9	80.7	326.7	167	58.7	180	-	240	84.6	260
odd	27	n-heptacosane	380.73	323.1	7.30	2.78	8.60	326.3	69.3	26.4	80.9	331.9	163	62.1	187	-	240	91.3	277
odd	29	n-nonacosane	408.79	326.0	7.10	2.90	8.90	331.4	73.3	30.0	90.4	336.5	166	67.7	201	-	239	97.6	300

2 Comparison with literature data – selected examples

To show that the new observations made here, which add details and also reveal significant deviations compared to existing literature, require the specific data set from high-accuracy and high-resolution calorimetry, a few examples are now given.

The most direct way to observe the difference is to look directly at *H* versus *n* and *S* versus *n* figures. The following figures show what the corresponding figures would look like if the data set from the previous publication [7] would have been used. These data are originally from Dirand et al. [15], Kahwaji et al. [16], and Briard et al. [17].

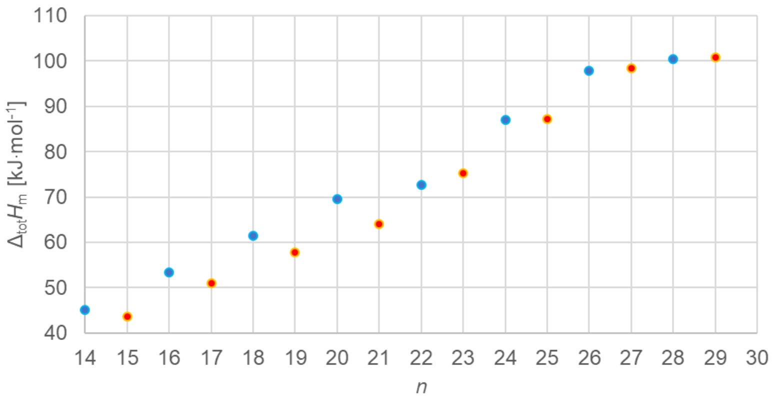

Figure S3: Corresponding to figure 12, using the data basis from [7].

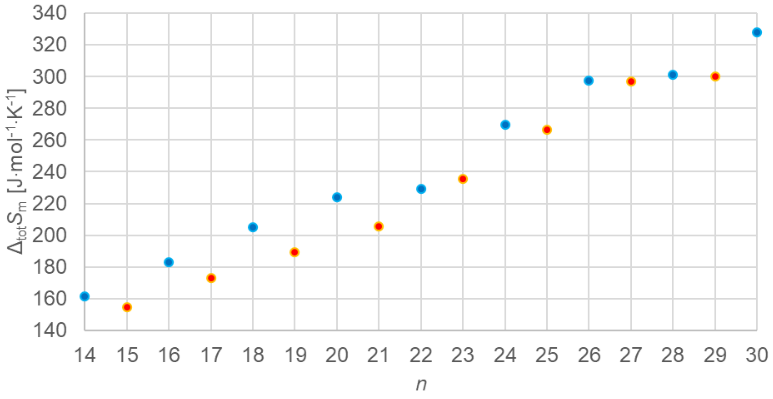

Figure S4: Corresponding to figure 13, using the data basis from [7].

Figures S3 and S4 show that, using the data basis from [7], the correlations in figures 12 and 13 can be guessed, however not more. The situation becomes even clearer when looking at a broader data basis. Faden et al. [18] analyzed literature data for octadecane, $n = 18$, and found for purity ≥ 99 % melting enthalpy values from AC measurements of 241.3 J/g, 242.5 J/g, and 236.5 J/g, while those from DSC measurements range from 220.4 J/g to 256.7 J/g. With the molar mass of 254.49 g/mol, the values are thus for the AC measurements within 2% of the value used here, while for DSC measurements they range from about 9 % below (56 J/mol) to about 6 % above (65 J/mol). For lower purities the deviations range even wider.

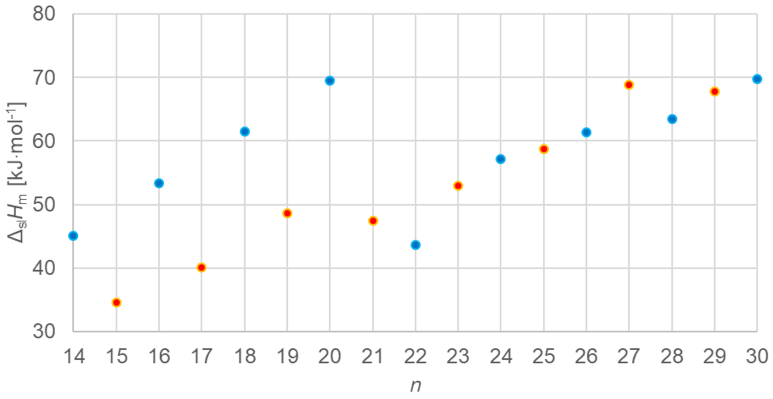

Figure S5: Corresponding to figure 14, using the data basis from [7].

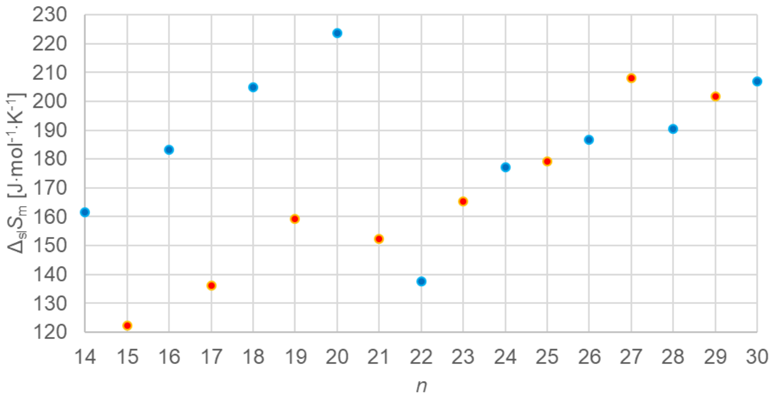

Figure S6: Corresponding to figure 15, using the data basis from [7].

Figure S5 and figure S6 correspond to figures 14 and 15 of the main manuscript, again now using the data basis of [7]. Without high-accuracy and high-resolution calorimetry data now the separate correlations between odd *n*, and between even *n* from 22 to 30, are not observed at all, thus showing the merit of the present study.

3 References

[7] Mehling, H.; White, M.A. Analysis of trends in phase change enthalpy, entropy and temperature for alkanes, alcohols and fatty acids. *Chemical Physics Impact 2023*, *6*. https://doi.org/10.1016/j.chphi.2023.100222.

[8] Mehling, H.; Leys, J.; Glorieux, C.; Thoen, J. Potential new reference materials for caloric measurements on PCM. *SN Applied Sciences* **2021**, 3, 202 | https://doi.org/10.1007/s42452-020-03929-y.

[11] Thoen, J.; Leys, J.; Losada-Pérez, P.; Glorieux, C. Enthalpy measurements of condensed matter by Peltier-element-based adiabatic scanning calorimeter (pASC). In *Enthalpy and Internal Energy: Liquids, Solutions and Vapours*; Editors E. Wilhelm and T. Letcher (RSC Publishing, Croydon, UK, 2018) pp. 77-95 (Chapter 3).

[13] Thoen, J.; Cordoyiannis, G.; Glorieux, C. High-resolution and high-accuracy calorimetry of order–disorder and melting transitions in the n-alkanes n-pentadecane, n-hexadecane, n-heptadecane, n-octadecane, n-nonadecane and n-eicosane. *J. of Chem. Thermodynamics* **2024**, *194*. https://doi.org/10.1016/j.jct.2024.107285.

[14] Leys, J.; Losada-Pérez, P.; Glorieux, C.; Thoen, J. Temperature Dependence of the Enthalpy of Alkanes and Related Phase Change Materials (PCMs). In *Enthalpy and Internal Energy: Liquids, Solutions and Vapours*; Editors E. Wilhelm and T. M. Letcher, The Royal Society of Chemistry 2018.

[15] Dirand, M; Bouroukba, M; Briard, A-J; Chevallier, V.; Petitjean, D.; Corriou, J-P. Temperatures and enthalpies of (solid + solid) and (solid + liquid) transitions of n-alkanes. *J Chem Thermodyn* **2002**, *34*, 1255–77. doi:10.1006/jcht.2002.0978.

[16] Kahwaji, S.; Johnson, MB.; Kheirabadi, AC; Groulx, D.; White, M.A. A comprehensive study of properties of paraffin phase change materials for solar thermal energy storage and thermal management applications. *Energy* **2018**, *162*:1169–82. doi:10.1016/j.energy.2018.08.068.

[17] Briard, A.-J.; Bouroukba, M.; Petitjean, D.; Hubert, N.; Dirand, M. Experimental Enthalpy Increments from the Solid Phases to the Liquid Phase of Homologous n-Alkane Series (C18 to C38 and C41, C44, C46, C50, C54, and C60). *J Chem Eng Data* **2003**, *48*, 497–513. doi:10.1021/JE0201368.

[18] Faden, M.; Höhlein, S.; Wanner, J.; König-Haagen, A.; Brüggemann, D. Review of Thermophysical Property Data of Octadecane for Phase-Change Studies. Materials 2019, 12, 2974; doi:10.3390/ma12182974

[19] Thoen, J.; Leys, J.; Glorieux, C. Adiabatic scanning calorimeter, European Patent EP 2 91328 B1 (22 September 2015), U.S. Patent No 9,310,263 B2 (12 April 2016).

[20] Leys, J.; Losada-Pérez, P.; Glorieux, C.; Thoen, J. Application of a novel type of adiabatic scanning calorimeter for high-resolution thermal data near the melting point of gallium, *J. Therm. Anal. Calorim.* **2014**, 117, 173. https://DOI 10.1007/s10973-014-3654-1

[21] Leys, J.; Losada- Pérez, P.; Slenders, E.; Glorieux, C.; Thoen, J. Investigation of the melting behavior of the reference materials biphenyl and phenyl salicylate by a new type adiabatic scanning calorimeter; *Thermochim. Acta* **2014**, *582*, 68–76, https://doi.org/10.1016/j.tca.2014.02.023

[22] NIST Chemistry WebBook, SRD 69. Available online: https://webbook.nist.gov/chemistry/name-ser/ (accessed between May and August 2024).

[23] VWR. Available online: https://de.vwr.com/store/product/738725/n-triacontan-99 (accessed on August 2024).
